# “How can I still be me?”: Strategies to maintain a sense of self in the context of a neurological condition

**DOI:** 10.3402/qhw.v9.23534

**Published:** 2014-04-11

**Authors:** Kerstin Roger, Monika Wetzel, Susan Hutchinson, Tanya Packer, Joan Versnel

**Affiliations:** 1 Department of Family Social Sciences, University of Manitoba, Winnipeg, Manitoba, Canada; 2 School of Health and Human Performance, Dalhousie University, Halifax, Nova Scotia, Canada; 3 School of Occupational Therapy, Dalhousie University, Halifax, Nova Scotia, Canada

**Keywords:** Chronic illness, neurological conditions, sense of self, strategies to maintain self-concept, biographical disruption, biographical flow

## Abstract

The Living with a Neurological Condition (LINC) study was part of the National Population Health Study of Neurological Conditions conducted in Canada. This article describes empirical original qualitative data collected in the third and final phase of this study and examines how individuals living with a neurological condition maintain continuity of their sense of self, with a particular focus on their strategies. Fifteen interviews were analysed for this paper. Emerging strategies for maintaining sense of self include: (1) avoidance and denial, (2) cognitive reframing, (3) articulation of the self through imagined positive identity, (4) strategies that reconnect to identity in the past, (5) adjusting and altering goals, (6) spiritual activities, (7) humour, (8) comparison with others: identity as shaped through social constructs, and (9) creating communities: a reciprocal reflection of self.

Chronic illnesses, which include neurological conditions, are known to have a strong impact on individuals who experience not only physical changes but also social, financial, mental, and spiritual changes. The Neurological Health Charities of Canada (NHCC, 2010) reports that not only will the rate of death and disability related to neurological conditions increase dramatically over the next 20 years, but our need to better understand the social and economic impact of these conditions will also rise significantly. A report on the burden of neurological diseases, disorders, and injuries in Canada by the Canadian Institute for Health Information (2007) suggested that neurological conditions represent one of the leading causes of disability in the Canadian population. Furthermore, as demonstrated by this report, few neurological conditions have a cure and most worsen slowly over years. Little research has been conducted in Canada on this population—in particular research that has collected original data of both a quantitative and qualitative nature. Furthermore, little is known about the strategies that individuals use to manage and sustain their sense of self in face of neurological illness. Since neurological conditions can affect individuals in many ways, this paper will focus on how 50 adult participants living with a neurological condition described strategies used to manage ongoing changes to their sense of self.

The data is derived from a national study (Living with a Neurological Condition or LINC) which utilized a variety of strategies in order to capture data from multiple sources and in multiple ways. The purpose of this study was to examine the impact of a neurological condition on the everyday lived experiences of Canadians, and the ability of health, social, and community services and agencies to support families and individuals to self-manage life with a neurological condition. The three nested studies included: Phase I, a quantitative population-based survey of adults (*n*=948) age 17 and older who have a neurological condition and parents (*n*=74) of children age 5–16 who have a neurological condition; Phase II, a quantitative study we called the “cohort study” including adults (*n*=125) and parents (*n*=18) of children with neurological conditions; and Phase III, a qualitative multiple perspective case study (MPCS) of 21 adults, six of whom were parents of children with neurological conditions and up to four of their selected supports. This paper utilizes qualitative data from this last MPCS phase of data collection. Given space limitations, additional detail about the process of all three phases of the study can be found elsewhere in a methods paper, because this paper only discusses data from the third phase (e.g., Versnel et al., do not present findings) (Versnel et al., 2013).

The terms of the funding received stipulated an emphasis on neurological conditions whose charitable organizations held membership in NHCC. These included: neurotrauma (e.g., acquired brain injury, brain tumour, spinal cord injury, and hydrocephalus); neuromuscular disorders (e.g., cerebral palsy, epilepsy, and spina bifida); degenerative demyelinating conditions [e.g., multiple sclerosis (MS)]; dystonia, and Tourette syndrome; and movement and other neurodegenerative disorders [e.g., or Guillain-Barre syndrome, Parkinson's disease, Huntington's disease, muscular dystrophies, dementia, and amyotrophic lateral sclerosis (ALS or Lou Gehrig's disease)]. Recruitment for the LINC study occurred through the national representative organizations of these groups, who distributed study related advertisements.

## Understanding the self

Turner (1976) argued that the self is a relatively stable, organized core of qualities that goes beyond self-esteem or idealized images through which people define themselves. The self consists of meaning (and meanings) that people attribute to themselves, that unify their subjective experiences over time, and that go beyond behaviours and actions (Denzin, 1986). People living with a chronic illness may experience a loss of self earlier in life than those without chronic conditions, with permeable boundaries of “who they are” accompanying changes related to illness, and new ways of understanding their sense of self emerging (Charmaz, 1983). As Charmaz (1997) suggests, people's self-concepts shift at different points in time throughout an illness, and this shift often occurs as a gestalt in relation to a previous view that life would generally be positive, progressive, and productive. According to Charmaz, illness can become an unwarranted attack on the self, and not just a recasting of self and self-concept.

In this light, it has been argued that the sudden onset of a chronic condition or illness may represent a piece of one's ongoing biography suggesting that despite threats to their identity, many aspects of their inner-self remain stable (Gelech & Desjardins, 2011) and continuous (Medved & Brockmeier, 2008) resulting in retained narratives of the self (Phinney, 2011). Faircloth and colleagues labelled this type of continuity “biographical flow” (Faircloth, Boylstein, Rittman, Young, & Gubrium, 2004). They suggest that rather than disrupting individuals’ biographies with the presence of certain social indicators, a chronic illness might be incorporated into their personal trajectory with other life events. Similarly, “biographical disruption,” a term introduced by Bury (1982), represents the emergence of uncertainty and interruptions in one's daily life due to the onset of chronic illness. Individuals come to face the burdens of their previously taken-for-granted world, and they must now rethink their self-concept, facing interruptions and inalterable changes to their personal identity as they carry on carrying on. Yet strategies that hope to and indeed do repair this disruption become very important.

In the literature are examples of strategies used to manage changes in sense of self. For example, some individuals do maintain existing activities while adjusting for their level of ability (Preston, Marshall, & Bucks, 2007). For others, a new diagnosis is integrated into their existing sense of self so that they can appreciate the abilities they still have and can see positive consequences emerging in their new situation (Sheldon, Renwick, & Yoshida, 2011). The literature describes that a “new normal” can be developed by some as a rubric for living well while also describing the incredible effort required to remain positive, hopeful, and well in the face of new disability (Driedger & Owen, 2008). As well, the ability to cope with others’ reactions and attitudes in order to conceal a diagnosis is not uncommon (Beard, 2004; Driedger & Owen, 2008). This current analysis examined strategies used by study participants living with neurological conditions to manage ongoing changes to their sense of self.

It is useful then to examine some of the strategies that people use that characterize how they manage ongoing changes to their sense of self in light of an illness; in other words, how they might attribute meaning to an established understanding of core attributes in light of new circumstances related to illness. A better understanding of such strategies would contribute to a wide range of possible clinical and practical applications: (1) improved education for the newly diagnosed in clinical and community based settings; (2) knowledge of alternative strategies made available to those already diagnosed; (3) lessons learned for staff providing support for clients; and (4) an improved understanding of how strategies can improve life circumstances and meaning of daily living, in a way that resists definitions of normalcy. With a new diagnosis or an ongoing neurological condition, the question is asked, “how can I still be me?”

## Methods

### Ethics

For all components of this study, ethical approval was received from the Health Canada Research Ethics Board, as well as the appropriate ethics review boards at Dalhousie University, Queens University, the University of Manitoba, and the University of Prince Edward Island. The province of Newfoundland ethics review board also acknowledged the study within that province.

### Multiple perspectives case study sample

The MPCS in Phase 3 of the LINC study was intended to provide an in-depth exploration from multiple perspectives to capture both uniqueness and complexity (Simons, 2009). MPCS is an example of a collective, instrumental case study (Stake, 2000). It is designed to maximize an understanding of the case rather than generalize from it. The participants for the MPCS were recruited from the cohort participants in the second phase of the LINC study, who themselves were recruited from the larger population survey. This population sample (Phase I) was recruited nationally through associated community based organizations as described above. Inclusion criteria for the cohort study were having at least one neurological condition or being a parent to a child with a neurological condition, being 17 to 65 years of age, and speaking English or French. All participants in the cohort study were informed about the third phase, the MPCS, and all participants in the MPCS were from Ontario or Atlantic, Canada. From the participants who volunteered, 21 adults were asked and agreed to participate as “focal participants” and are named either “parents” (their child is living with the condition) or “adults” (they are living with the condition) for the purposes of this study. These focal participants were recruited for variation in gender, condition, and diagnosis and were asked to identify up to six “nominated participants,” including at least one family member and one healthcare provider. No more than four per participant were contacted.

All MPCS focal participants were interviewed twice with some interviews occurring face-to-face at a place of their choice and with other interviews occurring by phone. An interview guide was used for the first interview, in that each person in the MPCS was asked these questions. For the second interview, the interview coordinator (a paid staff researcher with expertise in the area and working on the research team) constructed individualized interview guides which were open ended questions, in order to explore topics from the first interview in more depth or to clarify or gain focal participants’ perspectives on issues and topics discussed by nominated participants. All interviews were performed between June and October 2012. The interviews were recorded, transcribed verbatim, and checked for accuracy by the same research assistant who conducted the interview (Table I).

**Table I:** Summary of adult only focal participants.

Region	n	Gender	n	Age	n	Marital status	n	Employment	n
Atlantic Ontario	11	Female	10	20s	2	Married/common law	8	Full-time	4
	4	Male	5	30s	3			Part-time	3
				40s	6	Single	5	Retired	4
				50s	2	Divorced/separated	1	Long-term	3
				60s	2	Engaged	1	Disability	–
								Unemployed	0
								Student	1

### Data analysis

Interpretive description with its roots in nursing is particularly useful when investigating a clinical phenomenon and the goal of the research is implementation and change, rather than theory development (Thorne, 2008). Because interpretive description provides a basis for conceptual links between data and clinical practice, researchers applying this approach hope to locate particular experiences in a general context and to define their topic of interest within a personal or institutional process. What makes interpretive description so suitable to this study is that it allows for, and even encourages, an exploration of temporal and symbolic discussions between different sets of people, about health and illness, and with the possibility of clinical application.

Case-by-case analysis and cross-case analysis were conducted by applying Hsieh and Shannon's (2005) “directed content analysis” (p. 1281). They suggest that this includes applying analytic codes and categories that can be obtained from existing theories or literature, but it also includes expanding these upon further immersion in the raw data, and seeing which categories emerge and diverge. Overall, a range of quite rich categories were found in the MPCS data, which will be discussed in a series of papers. One of the foci of the LINC study was self-management. It became interesting as interviews proceeded to find out whether participants reflected on the topic of sense of self, and when we noted that they did through eventual open coding of MPCS interviews, this resulted in the creation of two initially quite broad categories: (1) a sense of self and identity, and (2) social impact on identity of condition. A second round of axial coding was conducted independently by two other researchers (Strauss & Corbin, 1998), revealing unique subcategories that were related to each other through their dimensions and properties: strategies specific to continuity and changes to the sense of self. The categories that emerged through the second round of coding will be discussed in the findings section of this paper. Finally, a third round of coding was conducted checking all adult focal participant interviews one final time to ensure categories at the second round of coding had not been left out, and that no other or new ones could be identified. Analyses for ethic themes were guided by the factors associated with the research objectives of the LINC study, the known literature on this topic, and the existing experience and expertise of the researchers involved (Table II).

**Table II:** Coding trajectory.

Round 1	Round 2	Round 3
Open coding: interview coordinator and author	Axial coding: two other researchers on LINC team	Selective coding: research assistant

### Rigor

Rigor was sought in ways established for content analysis and in qualitative research (Braun & Clarke, 2013). Trustworthiness of the outcomes was a goal that occurred through constant comparison of the data throughout the analytic process. An audit trail of the analysis, including memos along the way, was maintained throughout the analytic process and reviewed by co-authors in order to compare emerging categories and their relevant meaning. Because of the interdisciplinary nature of the team and the resulting diversity in investigator backgrounds and professional training, the investigators brought different perspectives to the data analysis minimizing the chance of bias from a single perspective or expertise base. The qualitative literature review completed in advance of coding contributed to the conceptual process in this paper. Attention was paid to discrepant data at several stages of the analysis by proposing alternative explanations or coming to an understanding of existing themes in the context of the literature review. Transferability was met through the cross-case format (Hsieh & Shannon, 2005). Key objectives of the study were taken into account throughout the data analysis, and surprising new themes or unexpected findings were discussed and presented.

## Findings

Nine themes emerged which are seen to be strategies to manage a sense of self for those living with a neurological condition. These are discussed as follows.

### Avoidance and denial

Participants spoke about quietly managing their personal emotions and thoughts about themselves, in light of what they were experiencing physically. This could mean disguising their physical pain from others, hiding their feelings of disappointment due to rejection by a long-standing friend, or saying nothing in order to protect a parent or a partner from their own real emotions. One of our participants, MF,^1^ described:

By the way, I denied it at first. If someone said, “Why are you limping?” I'd say, oh, I hurt myself running or something. It took about a year and a half before I just said, oh, I have MS. I mean it doesn't matter. Nobody … There's no relativeness to any of it. The relativeness is the physical. So when you're in a wheelchair that's when it matters, that you have something wrong with you.

This is an example of the way in which some participants in our study tried to keep their condition a secret, and in this process find space to work towards greater acceptance of their condition. Narratives about secrecy and avoidance tell us something about how our participants lived with change due to their illness and how this shaped their identity or their sense of self. Avoidance in the form of diversion, such as playing video games, watching TV, drawing, and listening to music, allowed participants to change the meaning of their situation when unable to change the reality of their condition.

### Cognitive reframing

Cognitively reframing how and what one thinks about oneself is another strategy participants in our study used to manage how they perceived themselves. AF mentioned,

Mentally, I try to be very independent even though physically I'm so dependent now. So I say that part, the mental and emotional doesn't have MS, but the physical I need help with and the rest I'm an equal.

AF articulated a sense of self that is constructed through both physical (the illness) and mental realities (“I try to be very independent”). However, she positioned the disease as an entity apart from her mental self: a strategy used to try to maintain a solid sense of self. She also said “I'm an equal”—equal to whom, one might ask? AF appeared to be making reference to an implicit normalcy, hinting at stigmatization that may come with disease, and suggesting that her core sense of self and identity could remain well. She provided us with a useful conceptual strategy, thinking of herself as equal in relation to others, and by doing so, she was moving away from seeing herself as sick and unwell, and seeing herself as worthy and equal.

### Articulation of the self through imagined positive identity

Our participants spoke about not wanting to be seen as a “sick person” or identified primarily through their diagnosis. As a result, participants chose to engage in “a lot of self-talk” to reflect upon and process the reality of their condition. MF said,

Everybody wants me to rest. I don't want to rest. Let's get on with it. I've got stuff to do here. I want to show you something. So that part is pretty good. Yes, I'm pretty fortunate. But my identity? I think it's changed but I'm still me. How I see myself has changed but I think the people that I care about, they still see me the same.

It is evident here that MF needed to redefine to others how she saw herself and her core identity (“I'm still me”). This may have been a response to the ways others saw her, a response to imagined or actual perceptions of others. This process of maintaining one's self-perception can be a very internalized and personal process, heightened by the invisible nature of many neurological conditions, especially when people are in the early stages and are not necessarily yet receiving the required support from others. Engaging in self-talk as a way to remain active was a way participants might establish and reinforce their feeling of “who I am.” In some cases, efforts were made to hold on to the positive aspects of life through self-talk, as GF (living with epilepsy and a brain injury) said, “Life goes on. Life is good.” In addition to recognizing positive aspects in one's own life, positive self-talk was also reflected in our participants’ attempts to distance themselves from the negative experiences associated with their condition. For instance, in hopes of being more positive, JF, a participant living with migraines and a brain injury, explained,

I'm finally getting to the point where ok, like … you know? … that's done and that's in the past [the accident that led to the brain injury] and let's just get on with my life now and see how … you know, just thinking about things to do ….

Here, JF is engaged in self-talk as seen by her words, moving herself forward into the future thinking about new goals and new activities.

#### Strategies that reconnect to identity in the past

Another way participants in our study attempted to maintain familiarity and a sense of self, despite new events related to their conditions, was by maintaining an identity that drew on long-term memory for perceived continuity of self. For instance, in JF's interview, while she expressed her relief to no longer be working in a stressful environment, she frequently referenced her success at her previous job. Reflecting on such long-term memories of her ability to “[work her way] up the ladder” at her place of employment to a well-paid position that would require a high level of expertise contributed to JF's sense of pride, which has continued to be a part of her identity long after the brain injury. In addition, even after being affected by her brain injury, JF was able to return to work, and can now reflect on this memory as a proof of a continued-self, making it easier for her to accept her condition. She stated,

I was glad to go back to work and … see all my friends and … you know and just to prove that ya it can be done, and I did it (laughs) … now I can say haha I did it … that's it and … we're done.

As Medved and Brockmeier suggest, memory importation represents one strategy which allows individuals to import memories from before their condition and transform them into their present reality to create continuity (Medved & Brockmeier, 2008). As a result, although JF is no longer working, she was able to draw on memories of working both before and after her diagnosis as “proof” of her abilities and overall success.

### Adjusting and altering goals

Continuity of a sense of self (“how can I still be me?”) was sometimes achieved when participants chose to remain engaged in, but alter or revise the goals of, the same types of activities they were engaged in before their diagnosis. As a result, in some cases, participants were able to adjust their goals by adapting the way they performed the activity. KF, who identified she was living with more than three of the designated conditions, explained,

I won't give [in] and get outside help as long as I can possibly do it because that's giving in to the disease and I won't, I'm not prepared to do that. So I still do housework, the housework isn't up to standard it once was, but I'm sure it passes.

In this quote, KF admitted to being unable to maintain the same standards of cleanliness that he used to identify with. However, by strategically altering his goal, KF was able to continue to engage in an activity in the face of his limitations giving him a sense of independence and control that is important to his sense of identity. For others, altering goals also involved substituting the activity that they could no longer complete for a similar activity. TF described his experience, where increasing limitations from MS meant that he was unable to continue the many physical activities that he once loved:

That probably impacts me more than anything […] What I can't do is to be as physically active as I used to be able to. Because everything else, you know, I can sort of do at moderation. I can't referee hockey, for example. Since the attack happened, since the disease happening, that's something I could never do. And I did it for 15 years. And so that's gone out of my life. And that impacts me mentally.

Being unable to engage in activities that were once a part of his daily routine were quite damaging to his self-concept and sense of self. However, TF was able to fill this void with his newfound passion for golf, stating,

… the only sport that I can play normally would be golf. And so there's the mental aspect that I play it. There's no limitation to anything because it's not a fast moving pace game anyway.

By replacing his lost activities with golf, TF advantageously distracted himself from his MS symptoms that manifest in other activities, while preserving his self-perception as a physically active man living a healthy lifestyle. Altering his goals became a key strategy. AF, an adult living with MS, saw this as one of her strategies as she explained,

So you know when my partner and I wanted to go to a concert but I can't go, then maybe my partner and I should rent a video or listen to something [at home], so I don't think about what I'm missing.

This participant could have said that she just became depressed, or felt sorry for herself doing nothing at all. Instead, she found an alternative to an otherwise desirable activity, as such reframing her goals.

### Spiritual activities

Some participants spoke of engaging in spiritually based activities as a way to maintain a sense of self. Those participants who talked about spirituality did so because of positive experiences with their own spiritual practices. For example, practicing different types of spiritual activities (the chakras), RF stated,

So I think it [spirituality] helps, from a spiritual aspect I think it helps because some beliefs say that you choose the life that you have, and I think that helps in terms of like, what lesson I'm trying to teach myself in this life. I think that's kind of neat, a neat way of looking at it …

Living with a chronic condition can often be unpredictable, and this can interfere with a person's daily activities and future planning. As this can be damaging to one's sense of self, RF, who is living with hydrocephalus, was able to utilize her spirituality to regain control of her future. GF stated that God was her “reason for living,” as her existence acts as evidence that “God still has a purpose for [her] life.” In this case, sustained spirituality gave her “a sense of direction” at a time in her life when so much is characterized by uncertainty, thus contributing to a preservation of her sense of self that she can rely on, as she knows that she will continue to have a purpose as long as she breathes.

In a longer explanation by CF, who stated she was living with more than three conditions, she also expressed the reassurance that her faith provided her, thereby solidifying her sense of past, present, and future self:

[My faith] keeps me calm in that I'm not afraid of death, I guess. It doesn't worry me at all. I firmly believe that there is something greater than me. Whether you want to call it God or Buddha or Allah or Jesus or whatever, there's something out there and there's something in me. And I guess my attitude is just like I'm going to believe what I want to believe. And when I die, I'll either be right or I'll be dead. So it won't matter. So it gives me courage to keep going and knowing that I might be here on this planet to do exactly what I'm doing.

### Humour

Another strategy to maintain a positive sense of self was using humour. Participants in this study would sometimes down play their challenges with the use of humour. HF, a participant with dystonia, referred to “just laugh[ing] it off,” and explained,

You know, I tried to tell them the story about, you know, how my family calls it [the spasms from the dystonia] Precious. I said, “Oh, Precious, this isn't a very good time right now.” You know? And I tried to … And they didn't get that humour.

In this example, HF reflected on having dystonia, and by using humour with her family, strategized how to bring people alongside her. Her sense of herself now includes this new character, a partner in crime called “Precious,” her dystonia.

In another interview, LF (living with MS and migraines) jokingly described her family's typical response to her reactions when she is tired. She stated,

They just put me to bed (laughs), “Moms cranky, it's time for her to go to bed.” Like, they know when I have to have a nap, they know when I have to go to bed, like that's just the way my body works.

### Comparison with others: identity as shaped through social constructs

In the face of limitations brought on by their conditions, participants compared themselves to others who were worse off than they as a strategy to manage their sense of self. KF explained,

This is just another cross to bear or something that goes with living and goes with dying, I just happen to be the unfortunate one, and it could be worse.

Later she also said,

I have to be like everyone else and tread water in the meantime and make the best of a blessed and wonderful experience, but I could have cancer you know and have all the problems a cancer patient might have,

Giving clear indication that she is aware that her condition is not the only one that exists. Through the acceptance of her condition, KF was able to reflect on her experiences in a way that acknowledges the obstacles in her life, while also putting her sense of self into perspective, as she is lucky relative to many other individuals.

### Creating communities: a reciprocal reflection of self

Our participants used old and new communities of support as a way to manage how they saw themselves and their sense of well-being as “I can still be me.” For example, CF suggested that Facebook had been an enormous resource since “ironically one of the people that's in the community that [I kept] most in touch with (on Facebook) ha[d] MS.” In addition to meeting new individuals with the same condition, MF and AF found their treatment team to be resourceful, as they respectively stated:

… when everyone's actually here, everyone's, I have a really good medical team, they're all really good […] so I have two physiotherapists, I have a few occupational therapists, just because one's not enough and a neurologist, physical medicine, a physiatrist, GP, dentist, ‘cause they're all part of it …

My need to be *cared for* has given me an excuse to spend time with a cast of varied and interesting characters. It is an opportunity to work intimately with people that would otherwise not cross my path.

In these situations, whether the companionship with participants is through sharing the same type of neurological condition or through professional relationships, participants stand to benefit from the added resource of information to help explain experiences related to their condition, as well as offer a sense of belongingness that they now have access to a community where they are not the anomaly. This sense of belonging is a new way of identifying themselves, that features of their lives are now recognizable as part of a new community—in a way that strengthens them and their sense of self and well-being.

## Discussion

The strategies discussed reveal the many ways in which the adult focal participants in this study managed a shifting sense of self in relation to their neurological condition. Not one of these strategies can be defined as negative, although each reveals the underbelly of some the participants’ daily challenges. The conceptualization of biographical flow and disruption presented in the introduction of this paper is valuable to how we understand a sense of self and the strategies used to maintain it. In this paper, the participants have demonstrated through their strategies for managing a sense of self that both biographical flow and disruption are an important part of their changing sense of self (Boeije, Duijnstee, Grypdonck, & Pool, 2002; Locock, Ziebland, & Dumelow, 2009; Penner & Roger, 2012; Roger & Medved, 2010). Diagnosis can be a moment where those diagnosed feel that their illness or injury has led them to develop a damaged identity, making them feel different or abnormal (Dickson, Barbour, Brady, Clark, & Paton, 2008). In turn, their outlook on life may change (Guise, McKinlay, & Widdicombe, 2010), especially when limitations from the condition interfere with the continuity of their communication skills (Dickson et al., 2008), interpersonal relationships (Dickson et al., 2008; Gelech & Desjardins, 2011; Roger, 2008), and even their “autonomy, mobility, daily activities, body, consciousness, and future goals” (Gelech & Desjardins, 2011, p. 66). Our participants revealed to us that sometimes avoiding a situation, or hiding it from others, was an essential skill that helped them get through the day and even feel good about themselves. At the same time, despite interruptions and disruption, maintaining and sustaining a sense of normalcy was central to the participants’ desire to manage their own daily lived experiences; defining “normal” shifted, however, and it changed depending on their experience of new or ongoing symptoms and changing disease trajectories. Our participants also revealed to us that humour helped them cope with responses from others and that seeing new strengths or new friendships as valuable was an important and positive shift.

These experiences suggest that biological disruption and repair to the sense of self may be an ever-changing and unstable reality, although *stable* in and of itself in the context of neurological decline. In fact, striving for some idea of normal and allowing that idea to shift was a constant effort for our participants, and it remained an essential aspect of how they managed their social and personal lives. This “state” of continuous change over time is a unique characteristic of those living with neurological decline, given the knowledge that there is no cure and that many conditions will worsen slowly and over a long period of time. In particular, knowing that changes to cognitive and mental aspects of health are also involved in these conditions, alongside physical and behavioural changes, shapes a unique future not comparable to other chronic conditions. Working towards a positive and strong sense of self in this context is a constant effort that attempts to establish balance between change and continuity, unpredictability, and the hope for a cure. When participants ask, “How can I still be me?” the answer lies in the abilities to use strategies in support of maintaining their sense of self.

In conclusion, it would be wrong to say that our participants experienced and spoke about only biological disruption or that they were always able to repair damage done to their sense of self; however, we can say that most participants demonstrated a remarkable breadth of cognitive and behavioural strategies that assisted them in not only managing their daily activities but, through this, also maintaining a strong and constantly changing, but positive, sense of self.

## Implications

The paper has discussed strategies that these participants used to better manage changes to their sense of self in light of living with a neurological condition. Understanding better how people might “see themselves” in relation to a sense of self, and the strategies they use to assist them to managing threats to their sense of self, will contribute to a better understanding of the daily lived experience of those living with neurological decline. The data have suggested that participants’ sense of self might be a silent yet critical factor in shaping how, when, and what activities they choose to (and can) participate in.

This knowledge can have clinical implications for professionals who want to assess and determine how best to keep those living with neurological conditions engaged in meaningful and useful activities; it can provide improved education opportunities for community staff and affected families, as well as individuals living with neurological conditions. Strategies can be used to further assist individuals in living full and rich lives despite their diagnosis.

Additional research on the link between sense of self and participation might include questions such as: How do individuals define a sense of self in relation to their chosen activities?; To what degree is this view of self linked to the activities they engage in, in relation to their condition?; How might these activities need new strategies with an understanding of a sense of self? In essence, given a new diagnosis or ongoing changes related to an existing one, future research might explore more explicitly “how can I still be me?” in relation to participation in valued activities.

## Limitations

The sample frame of the LINC study was not designed to capture a representative sample of all people in Canada with all neurological conditions. It is clear given the recruitment outcomes that people might have been underrepresented who belong to one of the following: those who did not belong to NHCC member organizations, and regional and local organizations who may not have had strong communication with their national representative organizations, people with severe communication or cognitive deficits, people without access to the Internet, and people with lower levels of education. In the cohort and MPCS sample, participants were limited to those under 65 and those living in Ontario and Atlantic provinces as well as not including those in institutional care. This paper focussed on an emerging question given an initial literature review conducted prior to data collection. Although self-management was a central topic for the LINC study, the topic of “self” or “sense of self” was not included as a key question in the study objectives or the MPCS interview questions and it emerged nonetheless in the interviews. Because qualitative interviews can result in topics that deviate or deepen an existing interview question, this paper demonstrates an emerging and interesting new area of inquiry.

## Footnotes

1 The authors decided not to provide more detailed information on individual MPCS participants, as it was found that the quotes might easily lead to identification in our context.
